# Combinatorial liposomal peptide vaccine induces IgA and confers protection against influenza virus and bacterial super‐infection

**DOI:** 10.1002/cti2.1337

**Published:** 2021-09-10

**Authors:** Mehfuz Zaman, Victor C Huber, Dustin L Heiden, Katerina N DeHaan, Sanyogita Chandra, Demi Erickson, Victoria Ozberk, Manisha Pandey, Benjamin Bailly, Gael Martin, Emma L Langshaw, Ali Zaid, Mark von Itzstein, Michael F Good

**Affiliations:** ^1^ Institute for Glycomics Griffith University Gold Coast QLD Australia; ^2^ Division of Basic Biomedical Sciences Sanford School of Medicine University of South Dakota Vermillion SD USA; ^3^ The Emerging Viruses, Inflammation and Therapeutics Group Menzies Health Institute Queensland Griffith University Gold Coast QLD Australia; ^4^ School of Medical Sciences Griffith University Gold Coast QLD Australia; ^5^ Global Virus Network (GVN) Centre of Excellence in Arboviruses Griffith University Gold Coast QLD Australia

**Keywords:** liposomes, modular vaccine, mucosal vaccines, multi‐pathogen, super‐infections

## Abstract

**Objectives:**

The upper respiratory tract is the major entry site for *Streptococcus pyogenes* and influenza virus. Vaccine strategies that activate mucosal immunity could significantly reduce morbidity and mortality because of these pathogens. The severity of influenza is significantly greater if a streptococcal infection occurs during the viraemic period and generally viral infections complicated by a subsequent bacterial infection are known as super‐infections. We describe an innovative vaccine strategy against influenza virus:*S*. *pyogenes* super‐infection. Moreover, we provide the first description of a liposomal multi‐pathogen‐based platform that enables the incorporation of both viral and bacterial antigens into a vaccine and constitutes a transformative development.

**Methods:**

Specifically, we have explored a vaccination strategy with biocompatible liposomes that express conserved streptococcal and influenza A virus B‐cell epitopes on their surface and contain encapsulated diphtheria toxoid as a source of T‐cell help. The vaccine is adjuvanted by inclusion of the synthetic analogue of monophosphoryl lipid A, 3D‐PHAD.

**Results:**

We observe that this vaccine construct induces an Immunoglobulin A (IgA) response in both mice and ferrets. Vaccination reduces viral load in ferrets from influenza challenge and protects mice from both pathogens. Notably, vaccination significantly reduces both mortality and morbidity associated with a super‐infection.

**Conclusion:**

The vaccine design is modular and could be adapted to include B‐cell epitopes from other mucosal pathogens where an IgA response is required for protection.

## Graphical abstract

In this study, we report on the potential of a modular multi‐pathogen mucosal vaccine. We found that this vaccine construct induces an Immunoglobulin A (IgA) response in both mice and ferrets. Vaccination reduces viral load in ferrets from influenza challenge and protects mice from influenza virus:*S*. *pyogenes* super‐infection.

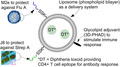

## Introduction

Influenza virus is a severe, acute respiratory pathogen with the capacity to generate strains capable of global pandemics. The Centers for Disease Control and Prevention (CDC) estimates that last year influenza was responsible for 24 000–62 000 deaths in the United States alone (https://www.cdc.gov/flu/about/burden/preliminary‐in‐season‐estimates.htm). Influenza A virus is the most important of the influenza virus types, causing alternate annual outbreaks and epidemics during the winter seasons in both hemispheres.^1^ However, many deaths are because of complications from secondary bacterial infections caused by *Streptococcus pyogenes, S. pneumoniae*, *Staphylococcus aureus* and *Haemophilus influenzae*. Secondary bacterial infections contributed significantly to the excess mortality observed during past influenza epidemics and pandemics.^2^ During the 2009 H1N1 influenza pandemic, 27% of all fatalities with laboratory‐confirmed influenza virus–bacterial co‐infections were found to be associated with streptococcus.^3^

Streptococcus alone also causes considerable loss of life. It is estimated to cause the loss of up to 500 000, mostly young, lives every year.^4^ In streptococcal‐endemic settings, autoimmune streptococcal‐related diseases (rheumatic fever [RF], rheumatic heart disease [RHD], post‐streptococcal glomerulonephritis) and invasive streptococcal disease (ISD) (often with toxic shock) are of major concern, particularly amongst disadvantaged populations.^5^ In Australian Aboriginal and Torres Strait Islander communities, for example, the rate of RF has been estimated to be as high as 651/100 000.^6^ In Alberta, Canada, from 2003 to 2017, the rate of ISD in First Nations people rose from 10.0 to 62.5 cases per 100 000. This is significant compared to a rise in the incidence of 3.7–8.2 cases per 100 000 in non‐First Nations people over the same period.^7^ In New Zealand, ISD is also most prevalent amongst Indigenous populations, who represent the most marginalised citizens in their country.^8^ RF and RHD are primarily linked to pharyngeal infections although in some settings there is an association between streptococcal pyoderma and RF.^9^

Highly effective vaccines to control both influenza virus and streptococcus are urgently needed. Currently, there are seasonal vaccines for influenza virus which demonstrate approximately 50% efficacy^10^, ^11^ and which require annual re‐immunisation with vaccines covering the prevalent circulating strains. Much effort has been directed to finding a universal influenza vaccine that does not require annual re‐editing. A promising target for such a vaccine is the transmembrane ion channel M2 protein.^12^, ^13^ The entire M2 protein ectodomain, or M2e, is a short peptide known to be highly conserved among human influenza viruses. Vaccination with M2e can confer protection against lethal challenge with H1N1, H3N2, H5N1 and H9N2 viruses in mice,^14^, ^15^ and it can reduce viral load in the respiratory tracts of both mice and ferrets.^16^

For streptococcus, different vaccine strategies are currently under development.^17^ The most advanced streptococcal vaccine is composed of 30 epitopes from the amino‐terminal region of the surface M protein.^18^ However, the coverage of this vaccine will be limited in streptococcal‐endemic areas where the extent of M‐protein diversity is significantly enhanced.^19^ A different approach focuses on the induction of antibodies to a conserved 12 amino acid M‐protein epitope, referred to as J8.^17^ Following vaccination with this epitope, antibodies can protect against multiple strains of streptococcus and passive transfer of J8‐specific antibodies can resolve invasive disease and toxic shock.^20^ Different vaccine delivery platforms have been used with this epitope, including peptide–protein conjugates delivered in Alum,^21^ the use of lipopeptides^22^, ^23^, ^24^ and delivery via proteasomes^25^; but the platform that has shown greatest efficacy against mucosal streptococcal disease was a liposome encapsulating diphtheria toxoid (to provide T‐cell help) with the J8 peptide attached by a fatty acid tail.^26^ This vaccine, delivered intranasally, induced mucosal IgA and protected mice from an intranasal streptococcal challenge.

IgA also plays a critical role in protection from influenza virus. Sridhar *et al.* ^27^ found that antibodies were the principal correlate of protection, with nasal IgA being shown to be the most critical factor determining protection in a human challenge model.^28^ Consequently, influenza A and streptococcal epitopes are very suitable candidates for inclusion in a multi‐pathogen liposomal vaccine designed to induce mucosal IgA. Inducing a simultaneous immune response to both pathogens would be ideal because as they both share a common seasonality^2^ and influenza virus co‐infection significantly exacerbates streptococcal‐associated morbidity and mortality.^2^, ^3^ Vaccination against influenza virus using a live or an inactivated vaccine was shown to only partially reduce mortality from an influenza virus:streptococcus super‐infection.^29^ In contrast, vaccination with a 6‐valent M‐protein vaccine protected all mice from death following an influenza virus:streptococcus super‐infection; however, all mice became ill following the influenza virus infection.^2^ Thus, a co‐vaccine containing highly conserved protective epitopes for both organisms should provide efficient protection against all strains of both pathogens and, in principle, significantly enhanced protection against a super‐infection. Furthermore, a co‐vaccine that does not require annual edits and re‐immunisations could provide long‐term improved respiratory health because of these pathogens.

Here, we extended the strategy employed to induce a protective J8 peptide‐specific mucosal IgA response to streptococcus by incorporating the conserved influenza A M2e epitope. We further incorporate 3D‐PHAD—the synthetic analogue of the Toll‐like receptor 4 glycolipid adjuvant monophosphoryl lipid A, known to be associated with induction of IgA.^30^ This modular liposome is a transformative vaccine design applicable to many respiratory mucosal pathogens, particularly where super‐infection is of great concern.

## Results

### Vaccination against the M2e peptide in a liposomal formulation elicits an IgA response and protection from influenza A virus challenge

**Figure 1:**
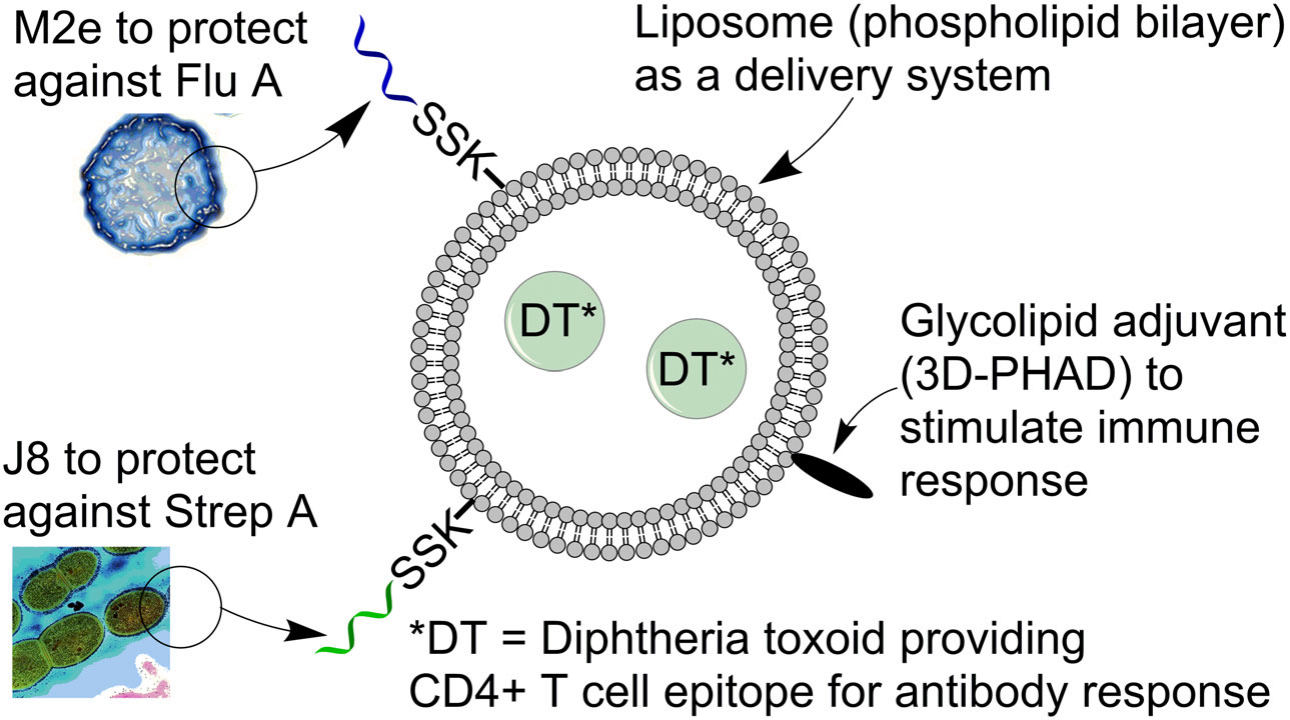
Idealised schematic of liposomal formulation. DT is encapsulated within neutral lipids and 3D‐PHAD inserted into the liposome membrane. To promote noncovalent complexing of peptide epitopes from streptococcus and/or influenza A virus to liposome bilayers, a hydrophobic anchor consisting of two palmitic acids (C16) is added to the epsilon and primary amine group of the lysine in a tripeptide spacer (consisting of Lys Ser Ser (KSS)), present in the amino‐terminus of peptide epitopes.

**Figure 2:**
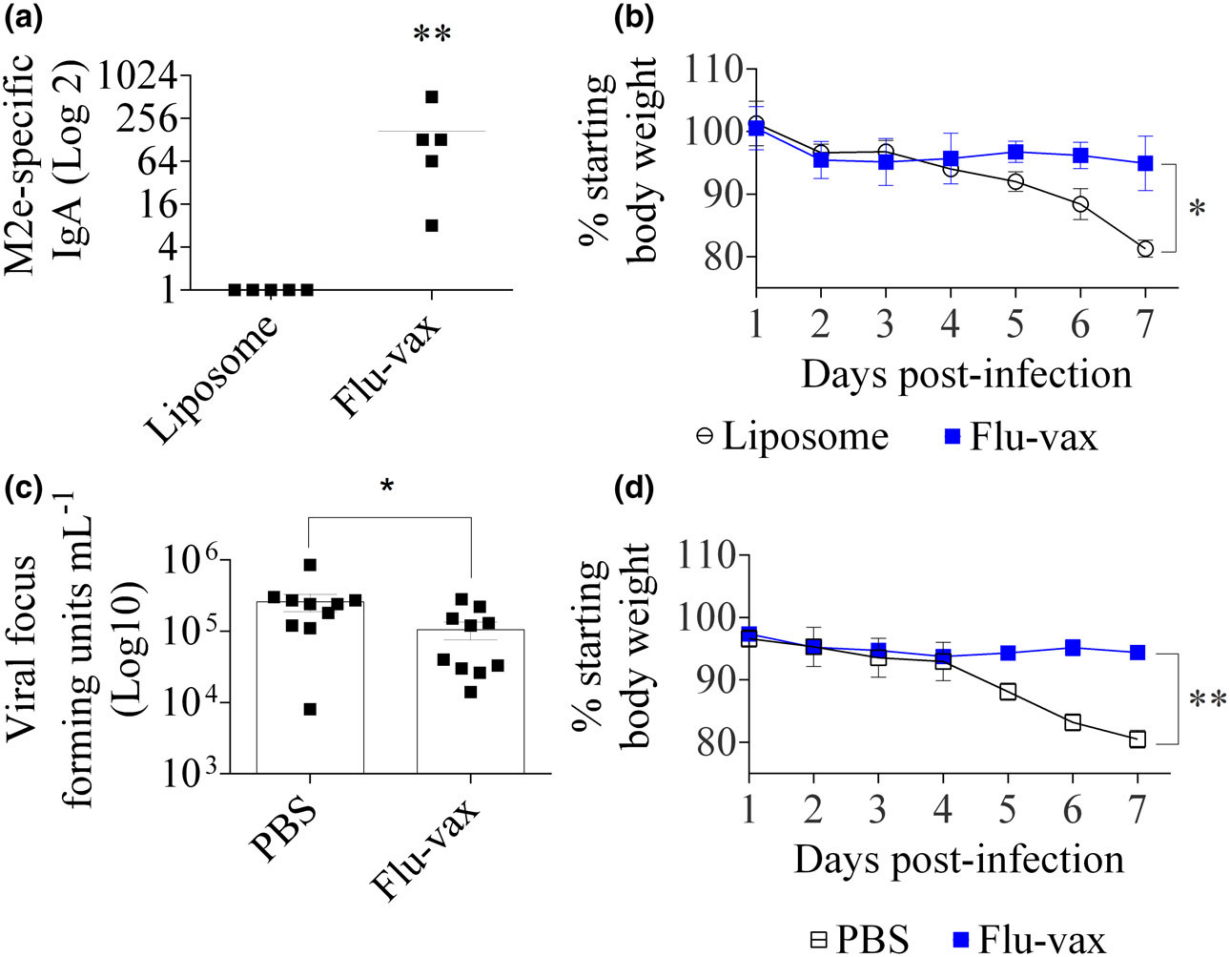
Vaccination of mice against the M2e peptide in a liposomal formulation elicits distinct immune responses and protection from influenza A virus challenge (data from two separate experiments are shown). **(a)** Mean mice (five per group) salivary IgA antibody titre. **(b)** Weight loss + SEM of mice (five per group) challenged with a sub‐lethal dose of PR8 influenza A virus. **(c)** In a repeat challenge study (ten mice per group) at days 4 and 7 after infection, five mice were euthanised, lung tissues harvested, and cumulative viral titres assessed by focus‐forming assay. **(d)** Weight loss + SEM of mice in the repeat challenge with a sub‐lethal dose of PR8 influenza A virus. Statistical significance (*, *P* < 0.05; **, *P* < 0.01; unpaired Mann–Whitney *U*‐test of test vs control).

We initially asked whether a liposomal vaccine expressing the M2e peptide would induce protection against influenza A virus. M2e expressed as a fusion protein with a proprietary hydrophobic protein was previously incorporated into liposomes and shown to induce protection following a combination of subcutaneous and intranasal immunisations^15^; however, the role of the hydrophobic protein and whether M2e, as a synthetic peptide in a liposome, would induce protection are not known. Importantly, induction of protective IgA antibodies following this vaccination scheduled was not measured in that study. Following intranasal vaccination with the M2e epitope in a liposomal formulation (Flu‐vax) (Figure 1), potent antigen‐specific salivary IgA antibodies were induced in mice (*n* = 5/group; Figure 2a). Immunised animals showed mild (insignificant) weight loss following viral challenge with the mouse‐adapted PR8 influenza A virus (A/PR/8/34 (H1N1)) at a sub‐lethal dose of 75 plaque‐forming units (PFU) (Figure 2b). Weight loss was significantly greater for the control group administered ‘empty’ liposomes (*P* < 0.05 on day 7; Figure 2b). In a repeat challenge study (*n* = 10/group), Flu‐vax immunised mice showed significantly reduced lung viral titres and body weight loss compared to phosphate‐buffered saline (PBS)‐immunised mice (Figure 2c, d).

**Figure 3:**
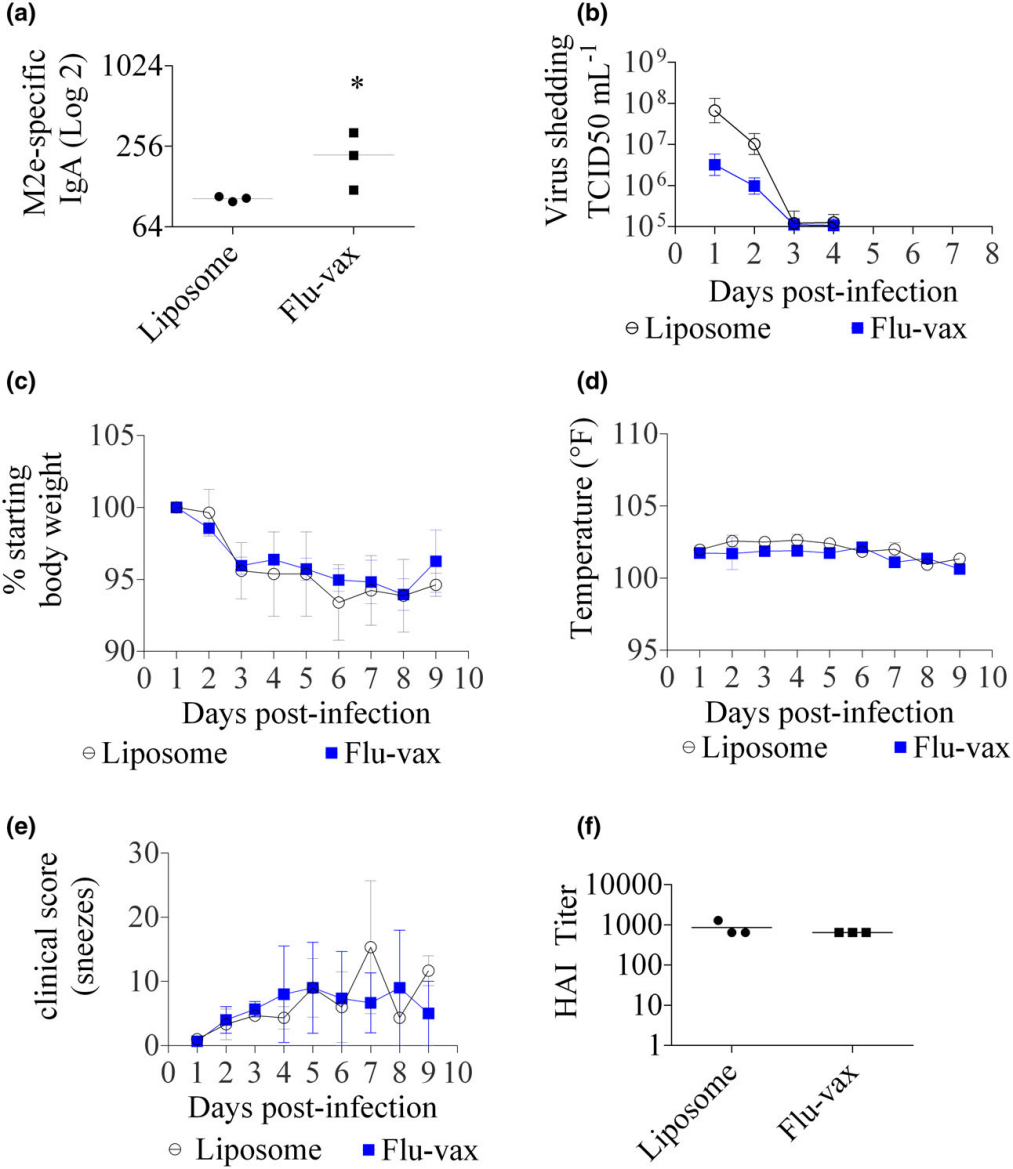
Vaccination of ferrets (three per group) against the M2e peptide in a liposomal formulation elicits distinct immune responses and protection from influenza A virus challenge in ferrets (results are from one experiment). **(a)** Mean ferret IgA antibody titre. Statistical significance (*, *P* < 0.05; **, *P* < 0.01; unpaired Mann–Whitney *U*‐test of test vs control). **(b)** Virus shedding from ferret respiratory tract after H1N1 influenza A virus challenge. **(c)** Weight loss of ferrets post‐infection. **(d)** Temperature of ferrets post‐infection. **(e)** Clinical score of ferrets post‐infection (changes in the ferret's level of sneeze activity were assessed). **(f)** Mean ferret post‐infection induced antibody response by HAI titre on Day 7 post‐infection.

Next, our vaccination strategy/formulation was assessed in ferrets, which are considered a more clinically relevant model because of their symptomatic susceptibility to influenza A infection.^31^ Vaccination with Flu‐vax (*n* = 3/group) induced significant salivary titres of M2e‐specific IgA (Figure 3a) and serum IgG (Supplementary figure 1). Antibodies were induced by day 21 and persisted until day 34 post‐immunisation when observations ceased (data not shown). Flu‐vax‐vaccinated ferrets that were challenged with the 2009 swine‐origin pandemic H1N1 influenza A virus (CA/4/09) showed > 90% reduction in viral titres in nasal secretions compared to the control animals vaccinated with ‘empty’ liposomes (Figure 3b). Cumulative virus shedding, when measured as area under the curve, was significantly lower in the Flu‐vax cohort post‐challenge compared to ferrets vaccinated with empty liposomes (*P* < 0.05 for days 1–8 using Stata software, version 13; data not shown). Post‐challenge weight, temperature and clinical score (combined sneeze activity) did not show significant differences (Figure 3c–e). Haemagglutination inhibition assay (HAI) results show that the ferrets were negative for antibodies against the virus envelope protein hemagglutinin (HA) on days 1–3 post‐infection (DPI) and all 6 ferrets seroconverted to the HA 7 days after infection (Figure 3f). Thus, in this non‐sterilising model,^16^, ^32^ Flu‐vax reduced viral load but did not affect the clinical signs of mild weight loss and sneezing.

### Vaccination with the J8 epitope and M2e epitope prevents death after influenza A virus:*S. pyogenes* super‐infection

**Figure 4:**
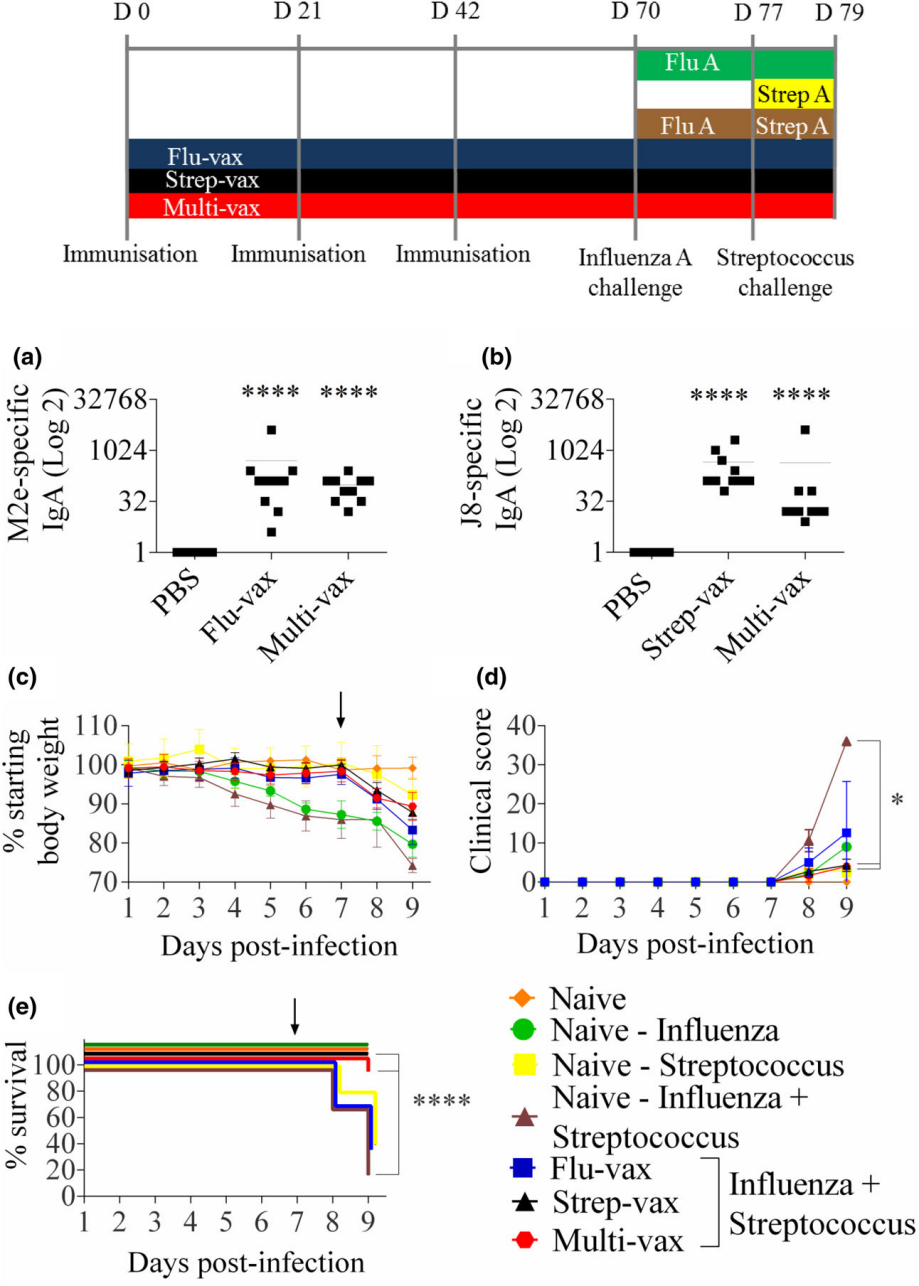
Vaccination against the J8 peptide and M2e peptide in a combined liposomal formulation prevents death after influenza virus:*S*. *pyogenes* super‐infection (ten mice per group; results are from one experiment). **(a)** Mean M2e‐specific mice salivary IgA antibody titre (the experiment was performed twice). **(b)** Mean J8‐specific mice salivary IgA antibody titre (the experiment was performed twice). **** indicates a significant difference between vaccinated and unvaccinated mice (*P* < 0.0001) using the unpaired Mann–Whitney *U*‐test of test vs control. **(c)** Weight loss + SEM of mice after super‐infection. The arrow within the figure represents the day of M1 streptococcus inoculation (day 7 after influenza A virus inoculation). * indicates a significant difference between vaccinated (Multi‐vax and Strep‐vax) and unvaccinated naïve – Influenza virus + Streptococcus control mice (*P* < 0.05) using the unpaired Mann–Whitney *U*‐test. **(d)** Clinical score of mice after super‐infection. **(e)** Survival after super‐infection. **** indicates a significant difference between vaccinated (Multi‐vax and Strep‐vax) and unvaccinated naïve – Influenza virus + Streptococcus control mice (*P* < 0.0001) using the log‐rank test.

**Figure 5:**
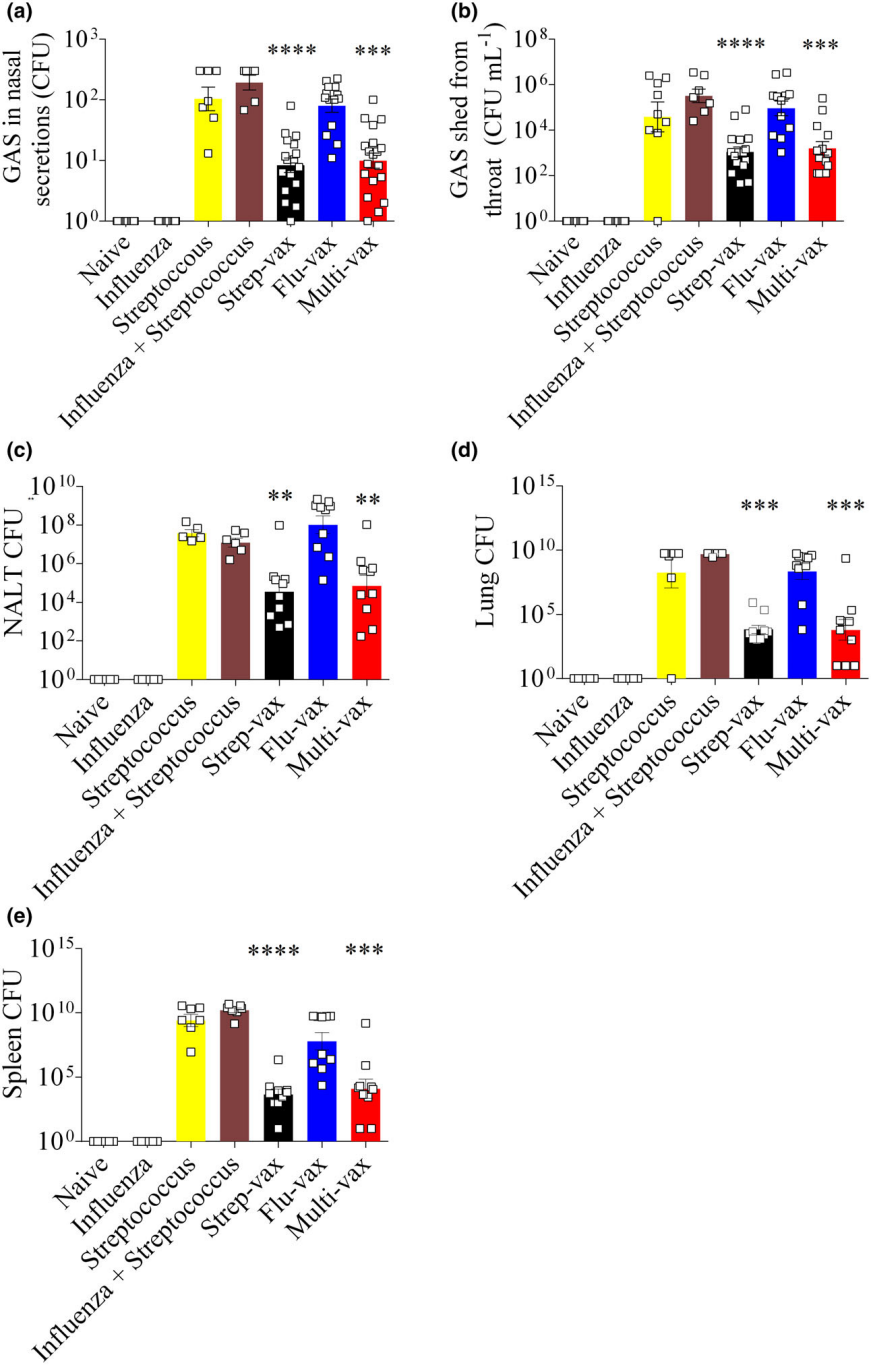
Reduction of bacterial bioburden post‐influenza virus:*S*. *pyogenes* super‐infection requires vaccination against the J8 peptide (ten mice per group; results are from one experiment). Bacterial burden results are represented as the mean CFU + SEM on days 8 and 9 for throat swabs and nasal shedding, and day 9 for all other samples. **(a)** Nasal shedding. **(b)** Throat swabs. **(c)** Colonisation of NALT. **(d)** Colonisation of lung. **(e)** Colonisation of spleen. Statistical analysis was performed using a nonparametric, unpaired Mann–Whitney *U*‐test to compare test groups to the Naïve – Influenza virus + Streptococcus control group (ns, *P* > 0.05; *, *P* < 0.05; **, *P* < 0.01; ***, *P* < 0.001).

To address whether (i) *S*. *pyogenes* super‐infection would lead to a significantly worse outcome and (ii) vaccination with a streptococcal vaccine, an influenza A virus vaccine or a combination vaccine would lead to improved outcomes, a number of vaccine candidates were explored. Thus, a vaccine containing both influenza A virus and streptococcal epitopes (‘Multi‐vax’, Figure 1) induced antigen‐specific mucosal IgA against both M2e and J8 at levels comparable to Flu‐vax and Strep‐vax given alone (Figure 4a, b). Vaccination also induced antigen‐specific serum IgG (Supplementary figure 2). Vaccinated and control mice were challenged intranasally with a sub‐lethal dose of PR8 influenza A virus (75 PFU) on day 0 (70 days post‐first dose of vaccine and 28 days after the final dose). Other unvaccinated mice were infected with either influenza virus (day 0), streptococcus pM1 (5 × 10^6^ colony‐forming units) (day 7) or both (days 0 and 7, respectively). Naïve, unvaccinated and unchallenged mice were used as an internal control. In unvaccinated mice, streptococcal infection alone caused ˜10% weight loss after two days (Figure 4c) and 60% mortality (Figure 4e). Mortality was likely because of high bacterial bioburden (Figure 5). Nevertheless, clinical scores were low (Figure 4d). However, a streptococcal super‐infection after mild influenza A virus infection led to a significantly worse outcome than either influenza A virus infection alone, or streptococcal infection alone, with the highest clinical scores greater than influenza virus (*P = *0.0152) or streptococcal infection (*P = *0.0667) alone, and 80% mortality (Figure 4e).

Mice that were vaccinated with either Strep‐vax or Multi‐vax had significantly improved outcomes. By day 7, naïve mice infected with influenza A virus had lost ˜13% of their body weight in comparison with naïve uninfected mice (*P = *0.0073; Figure 4c). Mice that had received either Flu‐vax or Multi‐vax had lost only ˜1–2% of their body weight in comparison with uninfected naïve mice (*P = *0.4178 and 0.4464, respectively; Figure 4c). We also observed mice that received Strep‐vax did not lose body weight in comparison with uninfected controls by day 7. All mice survived to this point. Following challenge with streptococcus, mice that had been vaccinated with Strep‐vax or Multi‐vax had significantly better clinical outcomes in terms of clinical scores (*P* < 0.05; Figure 4d) and survival (*P* < 0.0001; Figure 4e) in comparison with unvaccinated mice that underwent influenza A virus: streptococcus super‐infection.

Surprisingly, Strep‐vax prevented weight loss during the first seven days after infection with influenza A virus. We searched for any obvious sequence similarities between the streptococcal epitope and influenza A virus sequences that may explain how streptococcal vaccination could prevent weight loss because of influenza A virus infection. The sequence, KSSJ8, in Strep‐vax was found to have low identity with proteins from PR8 influenza A virus, as determined by pairwise protein sequence analysis (see the Supplementary Material in the Supporting file) using MultAlin software.^33^ We could not ascribe any obvious antigenic cross‐reactivity as the source of protection. We also considered that prevention of weight loss may have been because of a non‐specific effect of the Strep‐vax vaccine, as has been shown in other systems.^34^, ^35^, ^36^ However, vaccination with Lipo‐PHAD‐DT, without M2e or J8, did not reduce the viral load or prevent weight loss following influenza A virus infection (data not shown).

### Reduction of bacterial bioburden post‐influenza A virus:*S*. *pyogenes* super‐infection requires vaccination against the J8 peptide from streptococcus

To define the role of vaccination on bacterial outgrowth and systemic dissemination post‐super‐infection, organs and samples were assessed. Bacterial load in nasal discharge was significantly lower in mice immunised with either Multi‐vax or Strep‐vax compared to unvaccinated mice that were infected with either streptococcus or both influenza A virus and *S*. *pyogenes* (Figure 5a). Multi‐vax‐ and Strep‐vax‐immunised mice also showed significant protection against infection on the pharyngeal surface (Figure 5b), nasal‐associated lymphoid tissue (NALT) (Figure 5c), lungs (Figure 5d) and spleen (Figure 5e).

Mice that were vaccinated with either the streptococcal vaccine Strep‐vax or Multi‐vax had significantly elevated levels of CD4^+^ T cells, CD69^+^ activated CD4^+^ T cells and CD8^+^ T cells following super‐infection compared to unvaccinated mice (Supplementary figure 3).

Taken together, these data further extend the importance of vaccine‐induced immunity against *S*. *pyogenes* infection in the super‐infection model.^2^ Our data show that immunity against the streptococcal M protein‐derived J8 peptide reduces bacterial burden post‐influenza A virus*:S*. *pyogenes* super‐infection.

## Discussion

Here, we use animal models to show that different liposomal vaccines adjuvanted with the TLR4 agonist, 3D‐PHAD, and which express highly conserved peptide epitopes from either influenza virus or streptococcus, induce antigen‐specific IgA and protect against these pathogens. A vaccine expressing both peptide epitopes can protect against both pathogens. The epitopes individually have been shown to provide protection against multiple strains of influenza virus^13^ and streptococcus,^17^ but this is the first demonstration that a combined epitope vaccine can induce equivalent and efficient immunity against both pathogens. Importantly, the combined vaccine also provides significant protection against a super‐infection with both pathogens. The streptococcal component of the vaccine was shown to be the most critical component for protection against super‐infection. This likely relates to the challenge dose of influenza virus being low and non‐lethal, whereas the challenge dose of streptococcus resulted in 60% mortality in naïve mice. The modular nature of this vaccine platform means that epitopes from other respiratory pathogens can be easily included resulting in an epitope‐specific IgA response to all included epitopes. Although IgA has been shown to be important for protection against streptococcus^25^, ^37^ and influenza A virus,^28^ it has also been shown to be critical for protection against many other respiratory pathogens.^38^, ^39^, ^40^, ^41^

Streptococcal super‐infection following influenza A virus infection is a major concern. Over a quarter of fatalities reported in one study because of bacterial super‐infections in influenza patients were caused by streptococcus,^42^ while in another study, 7 of 10 patients with a streptococcal super‐infection died.^43^ Those who died had a median age of 37 years. In a separate study from France, one third of children with streptococcal infections also had laboratory‐confirmed influenza virus infections.^44^ Our animal model produced results consistent with these clinical findings. Twenty per cent of mice with a mild influenza virus infection that subsequently received a streptococcal infection survived. In comparison, 100% survival in mice with an influenza virus infection alone and 40% survival of mice that had a streptococcal infection alone was observed. While there was no difference in survival between mice that had a streptococcal infection alone and those with a super‐infection, there was a noticeable difference in clinical scores amongst surviving mice at day 9 (*P = *0.0667) and weight loss (*P = *0.0667). The difference in survival between *S*. *pyogenes*‐infected and super‐infected mice could not be attributed to differences in bacterial burden in any of the tissues tested (throat, NALT, lungs and spleen).

Our findings are consistent with data from other forms of streptococcus vaccines that overcome influenza virus‐mediated defects in host immune responses.^2^, ^42^ Immunity against streptococcus is an important component of protection against influenza virus*:*streptococcus super‐infection,^2^ ultimately protecting against secondary bacterial complications. However, we provide the first report of a vaccine against a super‐infection using conserved protective epitopes from both pathogens formulated within the same liposome.

Generally, super‐infection studies have evaluated the influenza virus‐mediated effects on host immunity that predispose towards death, using either *S*. *pneumoniae* or *S*. *aureus* as secondary invaders.^2^ Since *S*. *pyogenes* presents distinctive clinical diseases and has unique pathogenic properties,^45^ understanding its contribution to secondary infections is of substantial interest. We have demonstrated clearance of *S*. *pyogenes* in the URT tissue (throat and NALT) and nasal secretions following the challenge of liposome‐vaccinated mice.

Mucosal vaccines are an unmet clinical need as the majority of infections begin from mucosal surfaces including the URT. For many of these infections, including those associated with influenza virus:streptococcus super‐infections, there are currently no effective solutions. The present study addresses an important area in health sciences and, in particular, the lack of mucosal vaccine delivery systems for inclusion into new and novel vaccine formulations. It is likely that M2e‐based vaccines will not be a complete substitute for the currently available flu vaccines. However, the vaccine candidate investigated here provides a promising basis for the creation of a broadly protective flu vaccine together with other conserved antigens^46^ or supplemented with seasonal vaccines for broad and long‐lasting immune protection. This is the first study to utilise peptide epitopes from multiple pathogens in a single liposomal formulation to confer protection against both viral and bacterial pathogens. Since a strategy to incorporate both viral and bacterial antigens into a vaccine has not been previously pursued, the liposomal multi‐pathogen formulation described herein constitutes a significant development. This information will support more precise vaccine design and the development of novel anti‐infective therapeutics, accelerating medical countermeasure development against seasonal, pandemic influenza strains and secondary bacterial complications. This modular vaccine design approach could be readily adapted to include B‐cell epitopes from many other respiratory pathogens.

## Limitations of study

For challenge studies, the ferret and super‐infection experiments were performed once. While we have utilised established methods, repeat experiments would further substantiate the results. Extending the challenge experiments to other influenza virus and *Streptococcus pyogenes* strains to determine the extent of efficacy will be informative for future studies. This would highlight further the utility of the vaccine candidate.

## Methods

### Ethics statement

All animal protocols used were approved by the Griffith University Animal Ethics panel, GU Ref No: GLY/27/17/AEC. This study was done in accordance with the National Health and Medical Research Council (NHMRC) of Australia guidelines for the generation, breeding, care and use of genetically modified and cloned animals for scientific purposes (2007). Methods were chosen to reduce pain and distress to the mice, and animals were observed daily by trained animal care staff. Mice were terminated employing a CO_2_ inhalation chamber.

### Liposome formulation

For noncovalent complexing of J8 (QAEDKVKQ**SREAKKQVEKAL**KQLEDKVQ) or M2e (MSLLTEVETPIRNEWGCRCNDSSD) to the liposome bilayer, a hydrophobic anchor consisting of two palmitic acids (C16) was added to the epsilon and first amine group of the lysine present in a tripeptide spacer (consisting of Lys Ser Ser) present in J8 or M2e amino‐terminus (M2e‐KSS‐(C16)_2_ or J8‐KSS‐(C16)_2_). These constructs were manufactured by ChinaPeptides Co., Ltd (Shanghai, China). The expected mass of the construct (J8‐KSS‐(C16)_2_: MW 4061.97 g mol^−1^ and M2e‐KSS‐(C16)_2_: MW 3535.22 g mol^−1^) was confirmed by ESI‐MS and obtained at greater than 95% purity (by analytical RP‐HPLC area under the curve analysis). Liposomes were prepared using the thin‐film hydration method^47^ as described elsewhere.^26^ Neutral lipids from Avanti Polar Lipids, Inc. (Alabaster, AL, USA), were used at a molar ratio of seven dipalmitoyl‐sn‐*glycero*‐3‐phosphocholine (DPPC): two cholesterol (CHOL): one L‐α‐phosphatidylglycerol (PG). Lipids in chloroform (CHCl_3_) solution were coated onto round‐bottom flasks employing a rotary evaporator together with predetermined amounts of C16‐C16‐KSSJ8 or C16‐C16‐KSSS2. The volumes were 0.7 mL of DPPC (10 mg mL^−1^), 0.2 mL of CHOL (5 mg mL^−1^) and 0.1 mL of PG (10 mg mL^−1^). The resultant film of lipids was then dispersed in 1 mL of phosphate‐buffered saline (PBS) containing DT by vigorous mixing. The resultant liposomal suspension was centrifuged at 16 162 *g* for 10 min, the supernatant removed and the pellet resuspended in an appropriate volume of PBS to be freeze‐dried before administration.

### Freeze‐drying of liposomes

As established elsewhere,^26^ freshly prepared liposomes were freeze‐dried in 0.4 mL of Milli‐Q water (Merck Millipore; Burlington, MA, USA. 18.2 MΩ cm at 25°C) in glass vials with 10% trehalose (w/w). The vials were frozen in dry ice, dissolved in acetone for 10 min and placed on the plate of a freeze‐dryer with a temperature of −40°C. At the end of the freeze‐drying process, the glass vials were closed with a plastic cap and stored at 4°C.

### Intranasal immunisation of mice

BALB/c mice were anaesthetised by use of a mixture of xylazine and ketamine (1:1:10 mixture of xylazine: ketamine: H_2_O). Mice were administered formulations of Strep‐vax, Flu‐vax or Multi‐vax alone in a total volume of 20 µL PBS (10 µL per nare) while control mice were administered 20 µL of PBS (10 µL per nare). Two booster immunisations were given 21 days apart. Other control groups received equivalent amounts of liposome alone as described above.

### Serum and saliva sample collection

Serum was collected after primary immunisation to determine the level of J8‐specific systemic antibodies on days 20, 40 and 60. Blood collection via the tail artery was allowed to clot for 30 min at 37°C. Serum was collected after centrifugation for 10 min at 1000 *g*, heat‐inactivated for 10 min at 56°C and stored at –20°C.

Mice were administered 50 μL of a 0.1% solution of pilocarpine to induce salivation. Saliva was then collected in tubes containing 2 μL of 50 mmol L^−1^ phenylmethylsulfonyl fluoride (PMSF) protease inhibitor (Sigma‐Aldrich, St. Louis, MO, USA). Centrifugation for 10 min at 13 000 *g* was undertaken to remove particulate matter and samples stored at −80°C.

### Enzyme‐linked immunosorbent assay (ELISA) for antibody titres

Antigen‐specific serum IgG and mucosal IgA were measured as described elsewhere.^48^ J8 and M2e peptides were diluted to 0.5 mg mL^−1^ in carbonate coating buffer (pH 9.6) and coated onto polycarbonate plates in a volume of 100 μL/well overnight at 4°C. Unbound peptide was removed and blocked with 150 μL of 5% skim milk PBS‐Tween 20 for 2 h at 37°C. Following incubation, plates were washed 3 times with PBS‐Tween 20 buffer. Samples were serially diluted down the plate in 0.5% skim milk PBS‐Tween 20 buffer, starting at an initial dilution of 1:100 to a final dilution of 1:12 800 for sera and 1:2 to 1:256 for saliva samples. All samples were diluted to a final volume of 100 μL and incubated for 1.5 h at 37°C. The plates were washed 5 times and peroxidase‐conjugated goat anti‐mouse IgG or IgA (Invivogen, San Diego, CA, USA) were added at a dilution of 1:3000 or 1:1000, respectively, in 0.5% skim milk PBS‐Tween‐20 for 1.5 h at 37°C. After washing, 100 μL of OPD substrate (Sigma‐Aldrich) was added consistent with the manufacturer’s instructions and incubated in the dark for 30 min at room temperature. The absorbance was measured at 450 nm using a Victor^3^ 1420 multilabel counter (Perkin Elmer Life and Analytical Sciences, Waltham, MS, USA). The titre was defined as the lowest dilution that gave an absorbance of > 3 standard deviations (SD) above the mean absorbance of negative control wells (containing normal mouse serum immunised with PBS). Statistical significance (*P* < 0.05) was analysed using an unpaired Mann–Whitney *U*‐test to compare test groups to the PBS control group (*P* < 0.05 was considered significant) using GraphPad Prism 5 software (GraphPad, La Jolla, CA, USA).

### Procedure for influenza A virus:*S. pyogenes* super‐infection model challenge

The super‐infection model used for this study was described previously.^2^ Mouse‐adapted PR8 influenza A virus (A/PR/8/34 (H1N1)) was administered at a sub‐lethal and self‐limiting dose of 75 plaque‐forming units (PFU) on day 70 post‐primary immunisation. Weight loss (morbidity) and survival (mortality) were observed following influenza virus inoculation. After influenza virus inoculation, immunised and control mice were challenged intranasally with a predetermined dose of the streptococcus strain M1 on day 77 after primary immunisation. The *S*. *pyogenes* strain, pM1, had been serially passaged in mouse spleen to enhance virulence and made streptomycin‐resistant to enable streptococcus to be distinguished in throat swabs from normal murine bacterial flora.^49^ Morbidity (weight loss) was observed during the days after bacterial challenge and survival determined by blinded clinical scoring. Mice were culled because of high clinical scores using a monitoring sheet approved by the Griffith University Animal Ethics Committee. To determine streptococcus colonisation, throat swabs were obtained from mice on days 1 and 2 after challenge. The throat swabs were streaked out on Columbia blood agar (CBA; Thermo Fisher Scientific, Waltham, MS, USA) plates containing 2% defibrinated horse blood and incubated overnight at 37°C. Bacterial burden in nasal shedding was determined by pressing the nares of each mouse onto the surface of CBA plates ten times (single CBA plates/mouse/day) and exhaled particles were streaked out.^50^ On day 2 mice were culled, organ samples were homogenised in PBS, and samples were plated in duplicate using the pour plate method. For nasal shedding and throat swabs, results are represented as the mean CFU + standard errors of the means (SEM) for 10 mice/group on days 1 and 2. For organ samples, results are represented as the mean CFU + SEM for 10 mice/group on day 2. Differences in test groups versus the PBS control group were analysed with GraphPad Prism 5 using a nonparametric, unpaired Mann–Whitney *U*‐test (*P* < 0.05 was considered significant).

### Lung viral titre assessment

On days 4 and 7 after infection, BALB/c mice (*n* = 5 per group) were culled and their lung tissue removed and weighed. The lung tissue was homogenised and cleared by centrifugation for 10 min at 600 *g* and 4°C. The supernatant was taken and the viral titres were assessed using the influenza virus focus‐forming assay described below.

### Influenza A virus focus‐forming assay

Lung homogenate dilutions were used to infect MDCK cells and incubated at 37°C for 1.5 h, overlaid with Avicell (Sigma‐Aldrich) and incubated overnight at 37°C. Cells were fixed with 4% paraformaldehyde solution for 30 min and spots developed by *in situ* ELISA using an anti‐influenza virus NP‐antibody (Millipore MAB8257B; Merck Millipore), as previously published.^51^

### Ferret study to evaluate Flu‐vax

Young adult ferrets (Marshall Farms, Rose, New York, USA) were selected on the basis of low seroreactivity (haemagglutination inhibition titre, <1:40) against the circulating H1, H3 and B strains. Three ferrets/groups were continuously monitored for the body temperature of the ferrets. All procedures and experiments conducted on ferrets were performed within the biosafety level 2 facility in accordance with guidelines established by the animal care and use committee. Ferrets were administered formulations of Flu‐vax or liposomes alone in a total volume of 1 mL PBS (10 µL per nare). The ferrets received 2 booster immunisations on days 14 and 19 in the same fashion as the primary immunisation. On day 25, haemagglutination inhibition and ELISA (IgG and IgA) were carried out to confirm immune responses. On day 35 post‐primary immunisation, ferrets were challenged with the influenza virus (A/California/4/09‐H1N1) at 10^5^–10^6^ TCID_50_ dose in 1 mL volume via the intranasal route. After virus inoculation, all ferrets were monitored daily for signs and symptoms associated with weight loss and illness and were given a score using two individual criteria—activity and sneezing—in a manner similar to that reported by Reuman *et al.* ^52^ Activity and sneezing scores were recorded during a 10‐min period and were combined and reported as the clinical score for ferrets. Blood (1 mL) was collected via the internal mammary vein from isoflurane‐anaesthetised ferrets on the day of vaccination (day 0) and on days 4, 14, 21 and 34 after vaccination. Blood was also collected on days 1, 3, 5 and 7 post‐virus inoculation and was treated and used for serum antibody analysis.

On day 25 post‐primary immunisation and on days 1–8 after virus inoculation, ferrets were anaesthetised with 60 mg of ketamine (Hospira, Lake Forest, IL, USA), and nasal wash samples were collected as effluvium into 50‐mL tubes (Corning Incorporated, New York, USA) after the instillation of 1 mL of PBS (500 μL per nostril). For the determination of viral titres, virus was propagated in Madin‐Darby Canine Kidney (MDCK) cells. Confluent MDCK monolayers were rinsed with PBS and inoculated in quadruplicate with 10‐fold dilutions of nasal wash fluid. After 1 h, the inoculum was removed, and 1 mL of infection media supplemented with 1 μg mL^−1^ L‐(tosylamido‐2‐phenyl) ethyl chloromethyl ketone‐treated trypsin (TPCK‐trypsin; Worthington Biochemical, Lakewood, NJ, USA) was added to the cells. Cells were incubated for 4 days at 37°C in 5% CO_2_, and viral titres are reported as the TCID_50_ per millilitre.

### Flow‐cytometry procedure for analysis of circulating immune cells

Whole blood was collected from mice on days −1, 3, 7 and 9 post‐super‐infection. Blood was collected in 0.5 M EDTA, then lysed using ACK Lysis buffer, washed and resuspended in flow cytometry buffer (2% FCS + 0.5 mM EDTA in PBS). Single‐cell suspensions were blocked with anti‐CD16/32 antibody and stained with fluorochrome‐conjugated antibodies against mouse CD3 (clone 17A2; Biolegend, San Diego, CA, USA), CD4 (clone GK1.5, Biolegend), CD8 (clone 53‐6.2, BD Biosciences, Franklin Lakes, NJ, USA), CD45 (clone 30‐F11, BD Biosciences), CD11b (clone M1/70, BD Biosciences), IA/IE (M5/114; eBioscience, San Diego, CA, USA), Ly6G (clone 1A8, BD Biosciences), CD62L (clone MEL‐14, BD Biosciences) and CD69 (clone H1.2F3, BD Biosciences), and dead cells were excluded using LIVE/DEAD Near‐Infrared viability dye (#L110909, Thermo Fisher Scientific). Sphero™ Blank Calibration Particles (BD Biosciences) were added to the samples and used as reference counting beads. Cells were acquired on a BD LSR II Fortessa Cell Analyser, and data were analysed using FlowJo software (v10.6; Treestar, Inc.).

### Statistics

Analysis was performed using a nonparametric, unpaired Mann–Whitney *U*‐test (one‐tailed) to compare test groups to the PBS control group (ns, *P* > 0.05; *, *P* < 0.05; **, *P* < 0.01; ***, *P* < 0.001) with GraphPad Prism 5 software.

## Conflict of interest

The authors declare no conflict of interest.

## Author contributions

**Mehfuz Zaman:** Conceptualization; Data curation; Formal analysis; Funding acquisition; Investigation; Methodology; Project administration; Validation; Writing‐original draft; Writing‐review & editing. **Victor C Huber:** Conceptualization; Data curation; Formal analysis; Funding acquisition; Investigation; Methodology; Project administration; Resources; Supervision; Validation; Writing‐review & editing. **Dustin L Heiden:** Data curation; Formal analysis; Investigation; Methodology. **Katerina N DeHaan:** Data curation; Formal analysis; Investigation; Methodology. **Sanyogita Chandra:** Data curation; Formal analysis; Investigation; Methodology. **Demi Erickson:** Data curation; Formal analysis; Investigation; Methodology. **Victoria Ozberk:** Data curation; Formal analysis; Investigation; Methodology; Writing‐review & editing. **Manisha Pandey:** Project administration; Supervision. **Benjamin Bailly:** Data curation; Formal analysis; Investigation; Methodology; Resources; Writing‐review & editing. **Gael Gael Martin:** Data curation; Formal analysis; Investigation; Methodology. **Emma L Langshaw:** Data curation; Formal analysis; Investigation; Methodology; Writing‐review & editing. **Ali Zaid:** Conceptualization; Data curation; Formal analysis; Investigation; Methodology; Project administration; Writing‐review & editing. **Mark von Itzstein:** Conceptualization; Methodology; Resources; Supervision; Writing‐review & editing. **Michael Good:** Conceptualization; Funding acquisition; Investigation; Methodology; Project administration; Resources; Supervision; Writing‐review & editing.

## Supporting information

Supplementary figures 1‐4
