# Supplementary material for: Combinatorial liposomal peptide vaccine induces IgA and confers protection against influenza virus and bacterial super‐infection
Source: Clin Transl Immunology. 2021 Sep 10;10(9):e1337. doi: 10.1002/cti2.1337 (PMC8432089; doi:10.1002/cti2.1337)

Combinatorial liposomal peptide vaccine induces IgA and confers protection against influenza virus and bacterial super-infection

**Authors:** Mehfuz Zaman^1,*^, Victor C Huber^2,*^, Dustin L Heiden^2^, Katerina N DeHaan^2^, Sanyogita Chandra^2^, Demi Erickson^2^, Victoria Ozberk^1^, Manisha Pandey^1^, Benjamin Bailly^1^, Gael Martin^1^, Emma L Langshaw^1^, Ali Zaid^3,4,5^, Mark von Itzstein^1^ and Michael F Good^1,*^

**Supplementary figures**

**

**

**Supplementary figure 1.** Vaccination of ferrets (three per group; results are from one experiment) against the M2e peptide in a liposomal formulation elicits serum IgG antibody titer. Statistical significance (*, *P* < 0.05; unpaired Mann-Whitney *U*-test of test vs control).

**
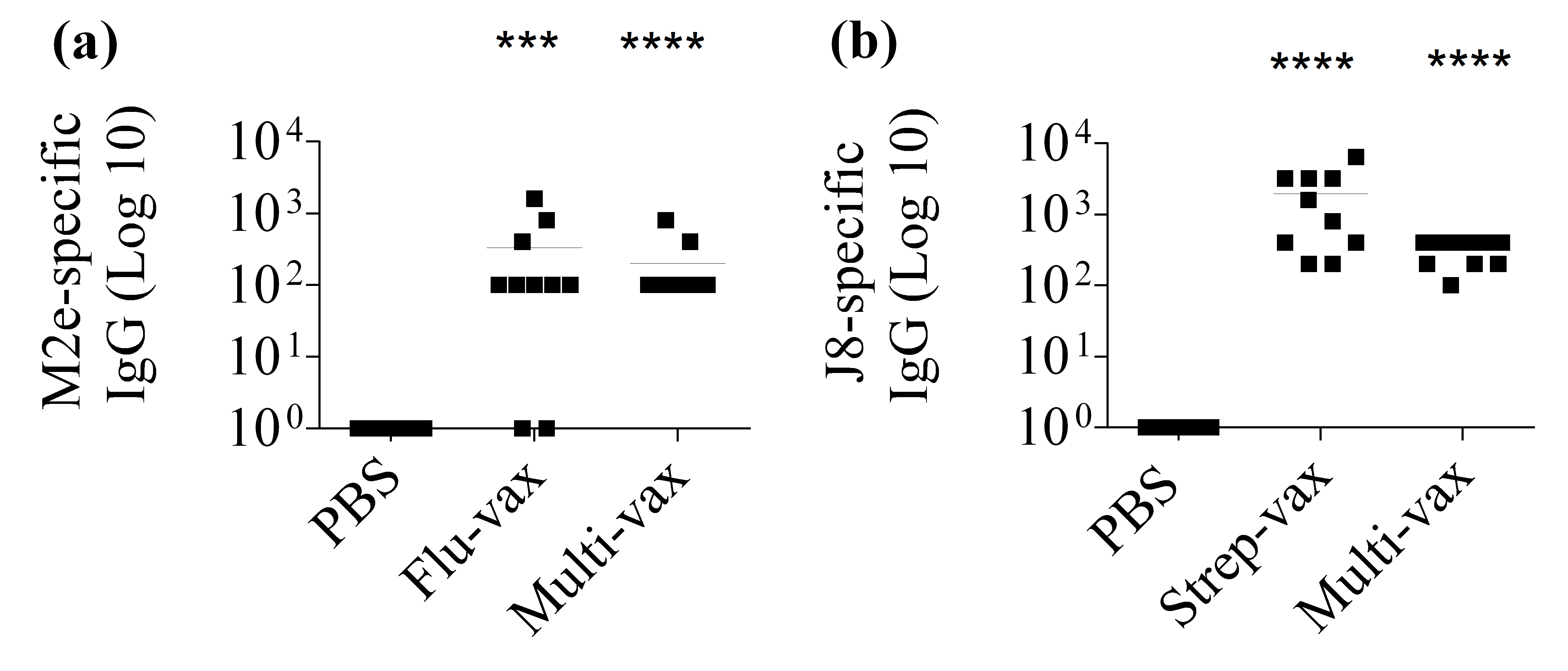
 Supplementary figure 2.** Vaccination of mice (ten per group; the experiment was performed twice) against the M2e/J8 peptides in liposomal formulations elicits IgG antibody titer. **(a)** Mean M2e-specific mice serum IgG antibody titer. **(b)** Mean J8-specific mice serum IgG antibody titer. Statistical significance (*, *P* < 0.05; unpaired Mann-Whitney *U*-test of test vs control).

**

**

**Supplementary figure 3.** Immunologic mechanisms post-super-infection demonstrates distinct responses by the multi-pathogen vaccine formulation (five to ten mice per group; results are from one experiment). The number of CD4^+^ **(a)**, CD69^+^ activated CD4^+^ **(b)**, CD8^+^ **(c)** and CD69^+^ activated CD8^+^ T-cells **(d)** was examined. Each symbol represents the mean ± standard error of mean from five to ten mice. Statistical analysis was performed by the non-parametric Mann–Whitney *t*-test (*, *P* < 0.05).

**
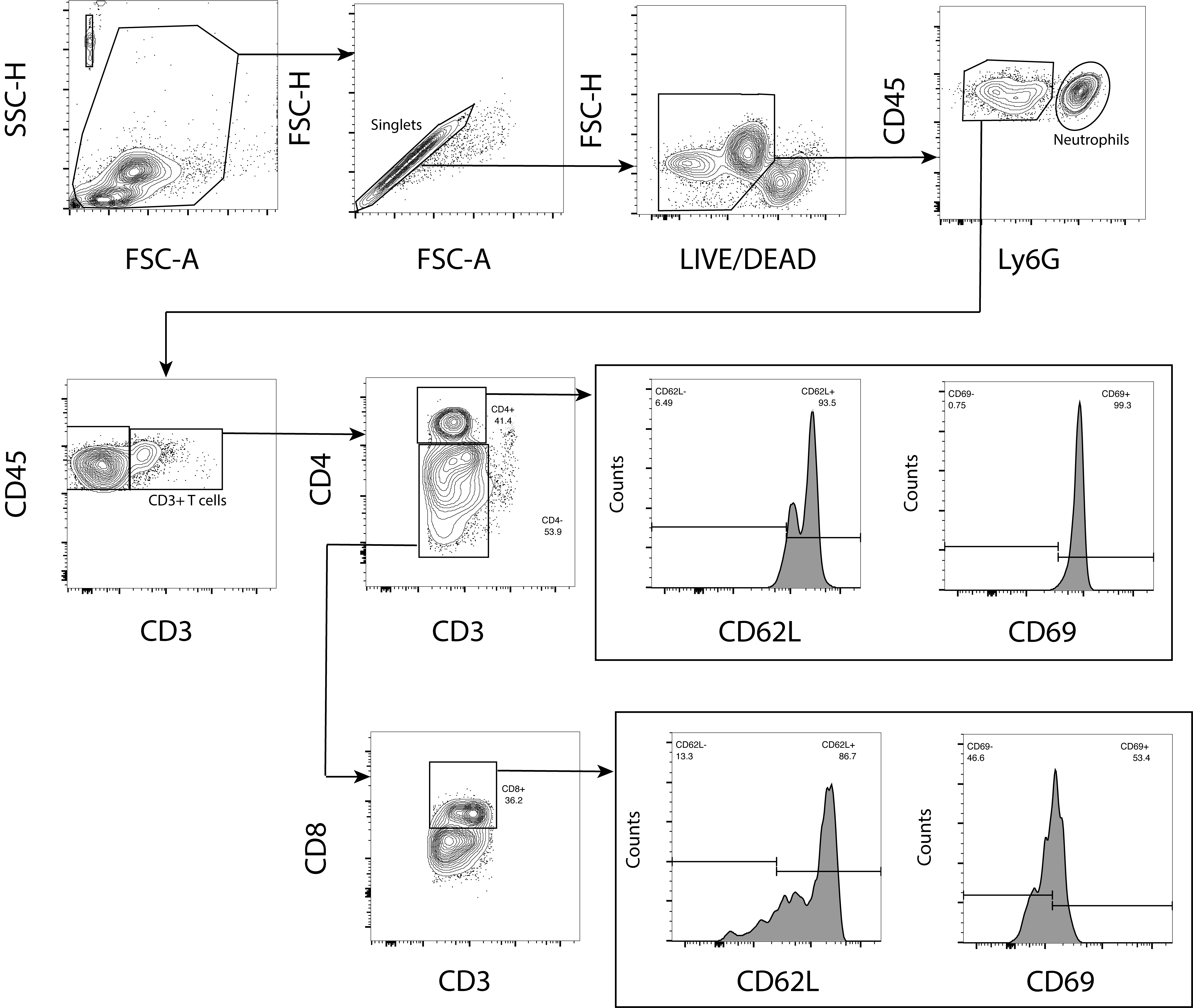
**

**Supplementary figure 4.** Flow cytometry gating strategy. The number of CD4^+^, CD69^+^ activated CD4^+^, CD8^+^ and CD69^+^ activated CD8^+^ T-cells from 5–10 mice were analyzed using flow cytometry on a BD LSR II Fortessa Cell Analyser. Flow cytometry data were analyzed using FlowJo software (v10.6; Treestar, Inc.).

**Supplementary Information**

**Sequence identity analysis between KSSJ8 and the PR8 influenza A virus proteins using UniProt knowledgebase (**[**http://www.uniprot.org/proteomes/**](http://www.uniprot.org/proteomes/)**).**

PR8 influenza A protein sequences were obtained from UniProt knowledgebase and each were aligned individually with the KSSJ8 amino acid sequence. The pairwise sequences were aligned using Mutalin software (<http://multalin.toulouse.inra.fr/multalin/multalin.html>).

- KSS-J8 is the top sequence
- Comparative protein is the middle sequence
- Consensus sequence is the bottom one - most likely sequence if they are the same
- Black = Amino Acid (AA) sequence (neutral)
- Red = high consensus - the same AA in both sequences
- Blue = low consensus - similar sequence, may be same type of AA or same AA family

**KSS-J8 and Matrix Protein 2**

>KSSJ8

KSSQAEDKVKQSREAKKQVEKALKQLEDKVQ

>Matrix2

MSLLTEVETPIRNEWGCRCNGSSDPLAIAANIIGILHLILWILDRLFFKCIYRRFKYGLKGGPSTEGVPKSMREEYRKEQQSAVDADDGHFVSIELE


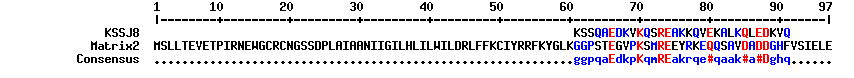


**KSS-J8 and PB2-S1**

>KSSJ8

KSSQAEDKVKQSREAKKQVEKALKQLEDKVQ

>PB2S1

MERIKELRNLMSQSRTREILTKTTVDHMAIIKKYTSGRQEKNPALRMKWMMAMKYPITADKRITEMIPERNEQGQTLWSKMNDAGSDRVMVSPLAVTWWNRNGPITNTVHYPKIYKTYFERVERLKHGTFGPVHFRNQVKIRRRVDINPGHADLSAKEAQDVIMEVVFPNEVGARILTSESQLTITKEKKEELQDCKISPLMVAYMLERELVRKTRFLPVAGGTSSVYIEVLHLTQGTCWEQMYTPGGEVRNDDVDQSLIIAARNIVRRAAVSADPLASLLEMCHSTQIGGIRMVDILRQNPTEEQAVDICKAAMGLRISSSFSFGGFTFKRTSGSSVKREEEVLTGNLQTLKIRVHEGYEEFTMVGRRATAILRKATRRLIQLIVSGRDEQSIAEAIIVAMVFSQEDCMIKAVRGDLNFVNRANQRLNPMHQLLRHFQKDAKVLFQNWGVEPIDNVMGMIGILPDMTPSIEMSMRGVRISKMGVDEYSSTERVVPLHQSKVECSSPHLL


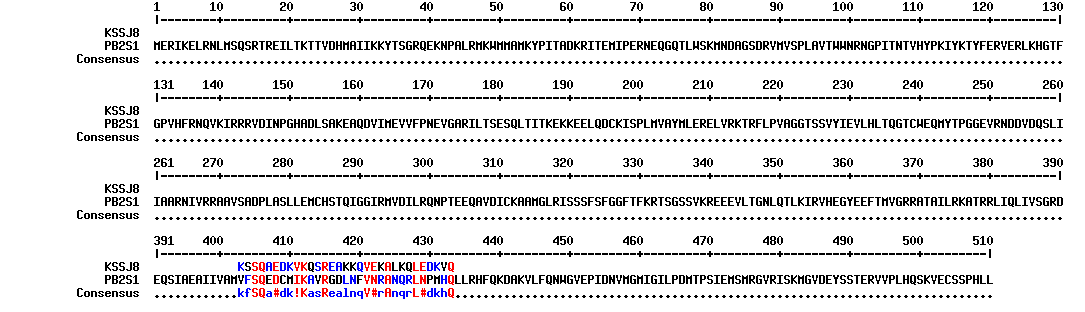


**KSS-J8 and POLYMERASE BASIC PROTEIN 2**

>KSSJ8

KSSQAEDKVKQSREAKKQVEKALKQLEDKVQ

>PolBasic2

MERIKELRNLMSQSRTREILTKTTVDHMAIIKKYTSGRQEKNPALRMKWMMAMKYPITADKRITEMIPERNEQGQTLWSKMNDAGSDRVMVSPLAVTWWNRNGPITNTVHYPKIYKTYFERVERLKHGTFGPVHFRNQVKIRRRVDINPGHADLSAKEAQDVIMEVVFPNEVGARILTSESQLTITKEKKEELQDCKISPLMVAYMLERELVRKTRFLPVAGGTSSVYIEVLHLTQGTCWEQMYTPGGEVRNDDVDQSLIIAARNIVRRAAVSADPLASLLEMCHSTQIGGIRMVDILRQNPTEEQAVDICKAAMGLRISSSFSFGGFTFKRTSGSSVKREEEVLTGNLQTLKIRVHEGYEEFTMVGRRATAILRKATRRLIQLIVSGRDEQSIAEAIIVAMVFSQEDCMIKAVRGDLNFVNRANQRLNPMHQLLRHFQKDAKVLFQNWGVEPIDNVMGMIGILPDMTPSIEMSMRGVRISKMGVDEYSSTERVVVSIDRFLRIRDQRGNVLLSPEEVSETQGTEKLTITYSSSMMWEINGPESVLVNTYQWIIRNWETVKIQWSQNPTMLYNKMEFEPFQSLVPKAIRGQYSGFVRTLFQQMRDVLGTFDTAQIIKLLPFAAAPPKQSRMQFSSFTVNVRGSGMRILVRGNSPVFNYNKATKRLTVLGKDAGTLTEDPDEGTAGVESAVLRGFLILGKEDKRYGPALSINELSNLAKGEKANVLIGQGDVVLVMKRKRDSSILTDSQTATKRIRMAIN


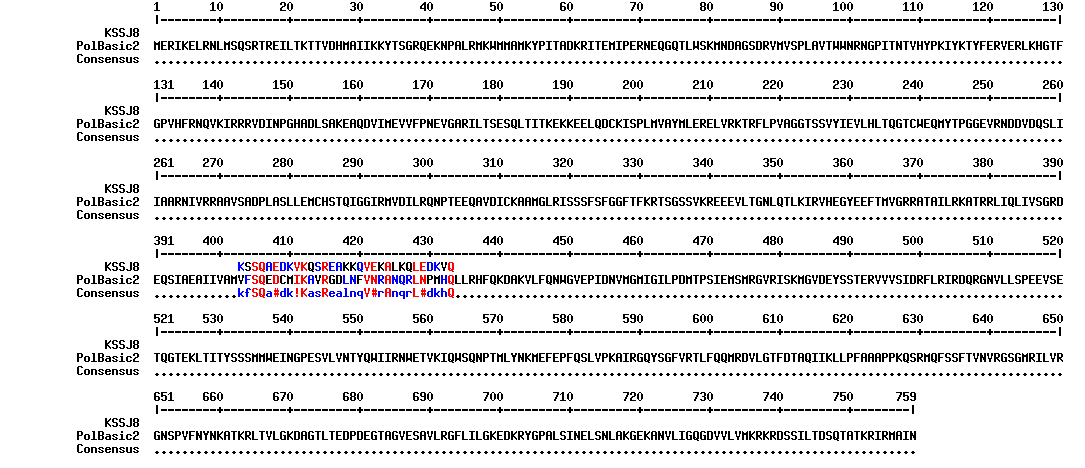


**KSS-J8 and Hemagglutinin**

>KSSJ8

KSSQAEDKVKQSREAKKQVEKALKQLEDKVQ

>Hemagglutinin

MKANLLVLLCALAAADADTICIGYHANNSTDTVDTVLEKNVTVTHSVNLLEDSHNGKLCRLKGIAPLQLGKCNIAGWLLGNPECDPLLPVRSWSYIVETPNSENGICYPGDFIDYEELREQLSSVSSFERFEIFPKESSWPNHNTNGVTAACSHEGKSSFYRNLLWLTEKEGSYPKLKNSYVNKKGKEVLVLWGIHHPPNSKEQQNLYQNENAYVSVVTSNYNRRFTPEIAERPKVRDQAGRMNYYWTLLKPGDTIIFEANGNLIAPMYAFALSRGFGSGIITSNASMHECNTKCQTPLGAINSSLPYQNIHPVTIGECPKYVRSAKLRMVTGLRNIPSIQSRGLFGAIAGFIEGGWTGMIDGWYGYHHQNEQGSGYAADQKSTQNAINGITNKVNTVIEKMNIQFTAVGKEFNKLEKRMENLNKKVDDGFLDIWTYNAELLVLLENERTLDFHDSNVKNLYEKVKSQLKNNAKEIGNGCFEFYHKCDNECMESVRNGTYDYPKYSEESKLNREKVDGVKLESMGIYQILAIYSTVASSLVLLVSLGAISFWMCSNGSLQCRICI


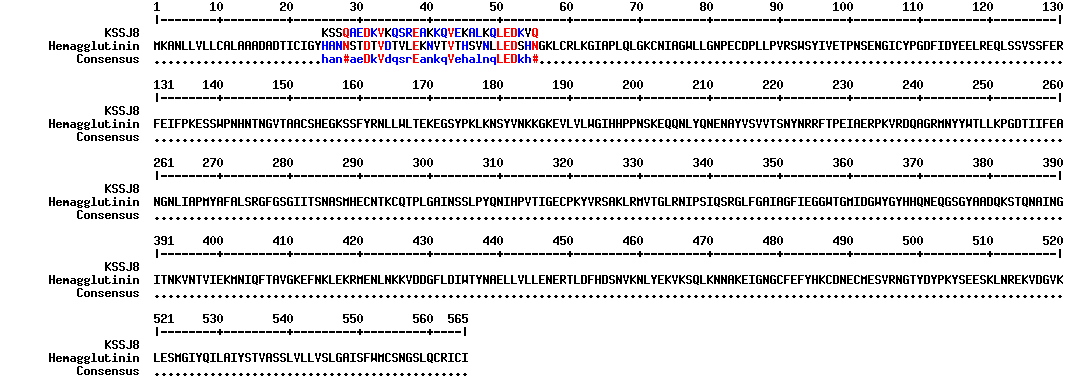


**KSS-J8 and RNA-directed RNA polymerase catalytic subunit**

>KSSJ8

KSSQAEDKVKQSREAKKQVEKALKQLEDKVQ

>RNApol

MDVNPTLLFLKVPAQNAISTTFPYTGDPPYSHGTGTGYTMDTVNRTHQYSEKGRWTTNTETGAPQLNPIDGPLPEDNEPSGYAQTDCVLEAMAFLEESHPGIFENSCIETMEVVQQTRVDKLTQGRQTYDWTLNRNQPAATALANTIEVFRSNGLTANESGRLIDFLKDVMESMKKEEMGITTHFQRKRRVRDNMTKKMITQRTIGKKKQRLNKRSYLIRALTLNTMTKDAERGKLKRRAIATPGMQIRGFVYFVETLARSICEKLEQSGLPVGGNEKKAKLANVVRKMMTNSQDTELSFTITGDNTKWNENQNPRMFLAMITYMTRNQPEWFRNVLSIAPIMFSNKMARLGKGYMFESKSMKLRTQIPAEMLASIDLKYFNDSTRKKIEKIRPLLIEGTASLSPGMMMGMFNMLSTVLGVSILNLGQKRYTKTTYWWDGLQSSDDFALIVNAPNHEGIQAGVDRFYRTCKLLGINMSKKKSYINRTGTFEFTSFFYRYGFVANFSMELPSFGVSGINESADMSIGVTVIKNNMINNDLGPATAQMALQLFIKDYRYTYRCHRGDTQIQTRRSFEIKKLWEQTRSKAGLLVSDGGPNLYNIRNLHIPEVCLKWELMDEDYQGRLCNPLNPFVSHKEIESMNNAVMMPAHGPAKNMEYDAVATTHSWIPKRNRSILNTSQRGVLEDEQMYQRCCNLFEKFFPSSSYRRPVGISSMVEAMVSRARIDARIDFESGRIKKEEFTEIMKICSTIEELRRQK


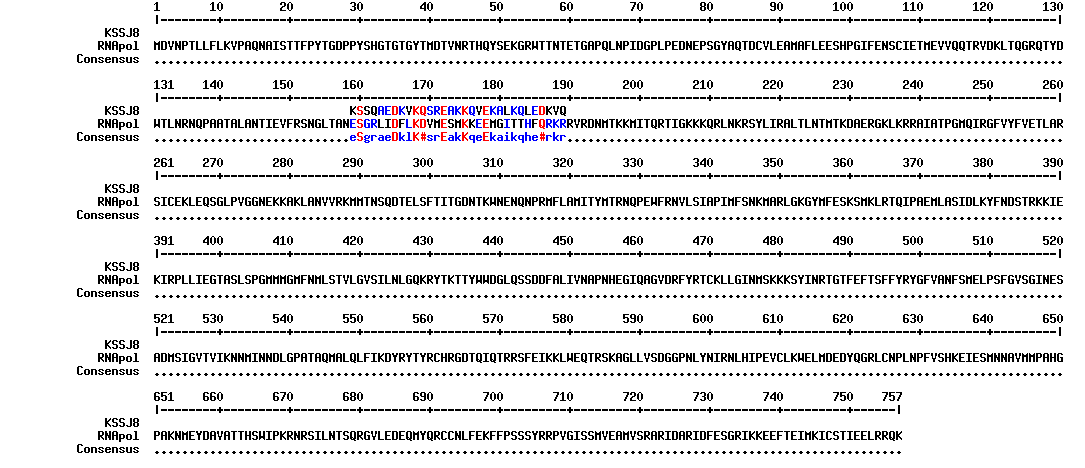


**KSS-J8 and Polymerase Acidic Protein**

>KSSJ8

KSSQAEDKVKQSREAKKQVEKALKQLEDKVQ

>PolAcidic

MEDFVRQCFNPMIVELAEKTMKEYGEDLKIETNKFAAICTHLEVCFMYSDFHFINEQGESIIVELGDPNALLKHRFEIIEGRDRTMAWTVVNSICNTTGAEKPKFLPDLYDYKENRFIEIGVTRREVHIYYLEKANKIKSEKTHIHIFSFTGEEMATKADYTLDEESRARIKTRLFTIRQEMASRGLWDSFRQSERGEETIEERFEITGTMRKLADQSLPPNFSSLENFRAYVDGFEPNGYIEGKLSQMSKEVNARIEPFLKTTPRPLRLPNGPPCSQRSKFLLMDALKLSIEDPSHEGEGIPLYDAIKCMRTFFGWKEPNVVKPHEKGINPNYLLSWKQVLAELQDIENEEKIPKTKNMKKTSQLKWALGENMAPEKVDFDDCKDVGDLKQYDSDEPELRSLASWIQNEFNKACELTDSSWIELDEIGEDVAPIEHIASMRRNYFTSEVSHCRATEYIMKGVYINTALLNASCAAMDDFQLIPMISKCRTKEGRRKTNLYGFIIKGRSHLRNDTDVVNFVSMEFSLTDPRLEPHKWEKYCVLEIGDMLIRSAIGQVSRPMFLYVRTNGTSKIKMKWGMEMRRCLLQSLQQIESMIEAESSVKEKDMTKEFFENKSETWPIGESPKGVEESSIGKVCRTLLAKSVFNSLYASPQLEGFSAESRKLLLIVQALRDNLEPGTFDLGGLYEAIEECLINDPWVLLNASWFNSFLTHALS


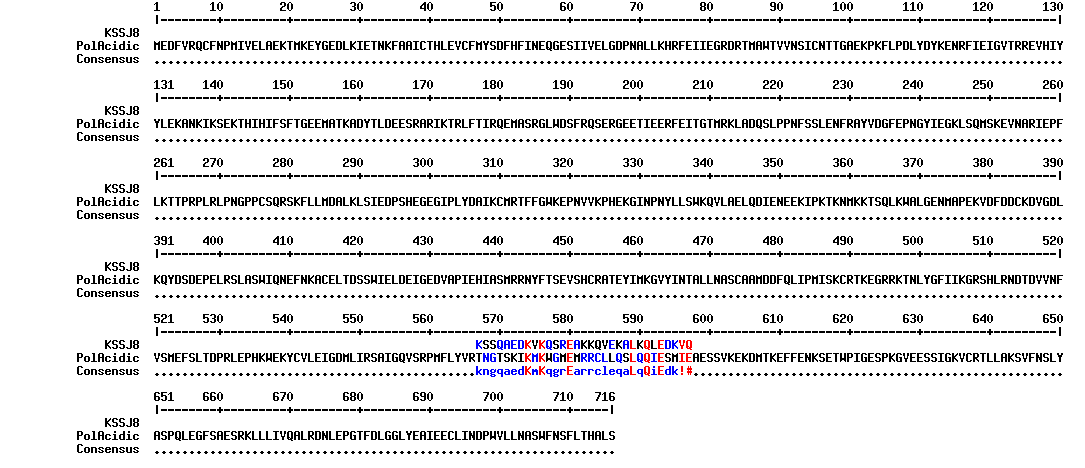


**KSS-J8 and Matrix Protein 1**

>KSSJ8

KSSQAEDKVKQSREAKKQVEKALKQLEDKVQ

>Matrix1

MSLLTEVETYVLSIIPSGPLKAEIAQRLEDVFAGKNTDLEVLMEWLKTRPILSPLTKGILGFVFTLTVPSERGLQRRRFVQNALNGNGDPNNMDKAVKLYRKLKREITFHGAKEISLSYSAGALASCMGLIYNRMGAVTTEVAFGLVCATCEQIADSQHRSHRQMVTTTNPLIRHENRMVLASTTAKAMEQMAGSSEQAAEAMEVASQARQMVQAMRTIGTHPSSSAGLKNDLLENLQAYQKRMGVQMQRFK


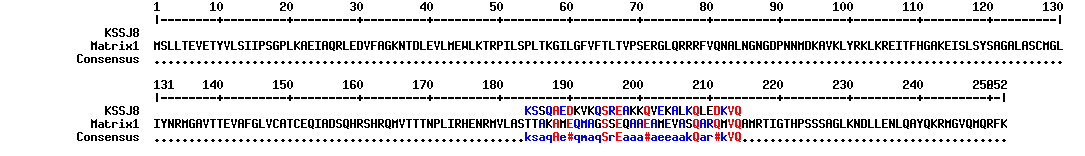


**KSS-J8 and Non-structural Protein 1**

>KSSJ8

KSSQAEDKVKQSREAKKQVEKALKQLEDKVQ

>NSProtein1

MDPNTVSSFQVDCFLWHVRKRVADQELGDAPFLDRLRRDQKSLRGRGSTLGLDIETATRAGKQIVERILKEESDEALKMTMASVPASRYLTDMTLEEMSRDWSMLIPKQKVAGPLCIRMDQAIMDKNIILKANFSVIFDRLETLILLRAFTEEGAIVGEISPLPSLPGHTAEDVKNAVGVLIGGLEWNDNTVRVSETLQRFAWRSSNENGRPPLTPKQKREMAGTIRSEV


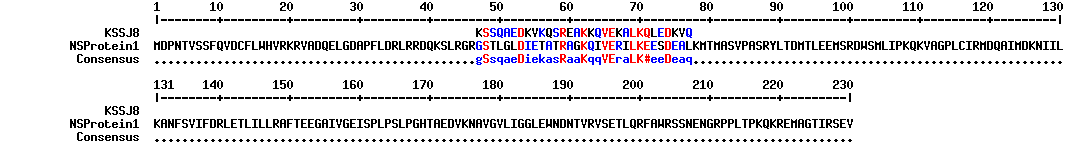


**KSS-J8 and Neuraminidase**

>KSSJ8

KSSQAEDKVKQSREAKKQVEKALKQLEDKVQ

>Neuraminidase

MNPNQKIITIGSICLVVGLISLILQIGNIISIWISHSIQTGSQNHTGICNQNIITYKNSTWVKDTTSVILTGNSSLCPIRGWAIYSKDNSIRIGSKGDVFVIREPFISCSHLECRTFFLTQGALLNDKHSNGTVKDRSPYRALMSCPVGEAPSPYNSRFESVAWSASACHDGMGWLTIGISGPDNGAVAVLKYNGIITETIKSWRKKILRTQESECACVNGSCFTIMTDGPSDGLASYKIFKIEKGKVTKSIELNAPNSHYEECSCYPDTGKVMCVCRDNWHGSNRPWVSFDQNLDYQIGYICSGVFGDNPRPEDGTGSCGPVYVDGANGVKGFSYRYGNGVWIGRTKSHSSRHGFEMIWDPNGWTETDSKFSVRQDVVAMTDWSGYSGSFVQHPELTGLDCMRPCFWVELIRGRPKEKTIWTSASSISFCGVNSDTVDWSWPDGAELPFSIDK


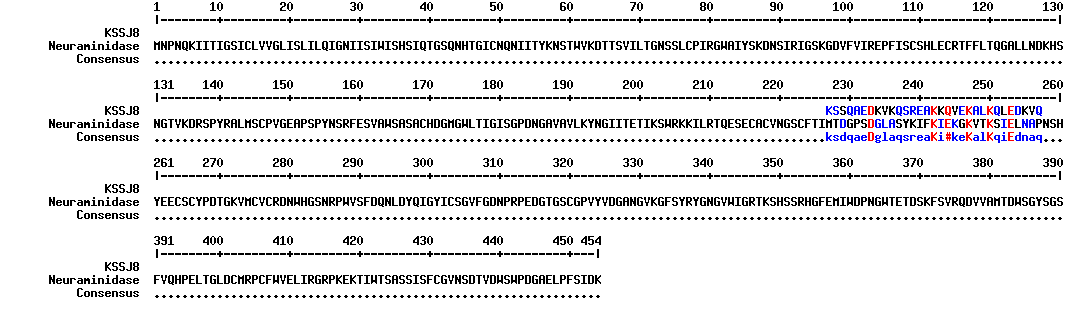


**KSS-J8 and Nuclear Export Protein**

>KSSJ8

KSSQAEDKVKQSREAKKQVEKALKQLEDKVQ

>NuclearExport

MDPNTVSSFQDILLRMSKMQLESSSGDLNGMITQFESLKLYRDSLGEAVMRMGDLHSLQNRNEKWREQLGQKFEEIRWLIEEVRHKLKITENSFEQITFMQALHLLLEVEQEIRTFSFQLI


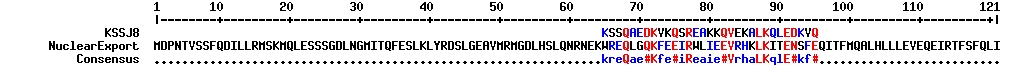


**KSS-J8 and Nucleoprotein**

>KSSJ8

KSSQAEDKVKQSREAKKQVEKALKQLEDKVQ

>Nucleoprotein

MASQGTKRSYEQMETDGERQNATEIRASVGKMIGGIGRFYIQMCTELKLSDYEGRLIQNSLTIERMVLSAFDERRNKYLEEHPSAGKDPKKTGGPIYRRVNGKWMRELILYDKEEIRRIWRQANNGDDATAGLTHMMIWHSNLNDATYQRTRALVRTGMDPRMCSLMQGSTLPRRSGAAGAAVKGVGTMVMELVRMIKRGINDRNFWRGENGRKTRIAYERMCNILKGKFQTAAQKAMMDQVRESRNPGNAEFEDLTFLARSALILRGSVAHKSCLPACVYGPAVASGYDFEREGYSLVGIDPFRLLQNSQVYSLIRPNENPAHKSQLVWMACHSAAFEDLRVLSFIKGTKVLPRGKLSTRGVQIASNENMETMESSTLELRSRYWAIRTRSGGNTNQQRASAGQISIQPTFSVQRNLPFDRTTIMAAFNGNTEGRTSDMRTEIIRMMESARPEDVSFQGRGVFELSDEKAASPIVPSFDMSNEGSYFFGDNAEEYDN


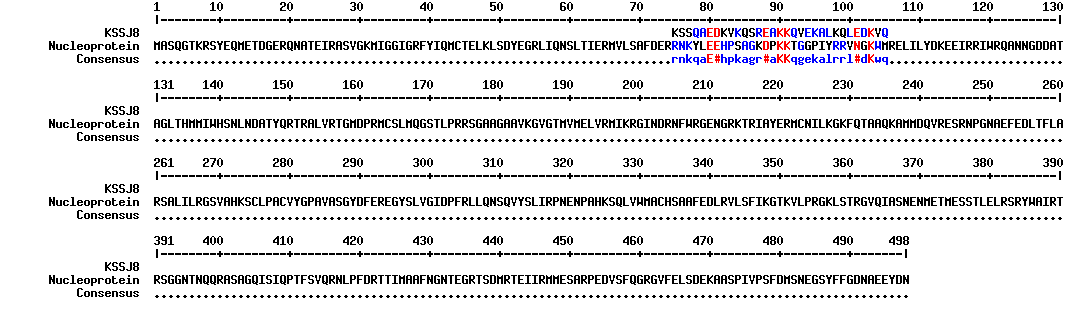


**KSS-J8 and Protein PB1-F2**

>KSSJ8

KSSQAEDKVKQSREAKKQVEKALKQLEDKVQ

>PB1F2

MGQEQDTPWILSTGHISTQKRQDGQQTPKLEHRNSTRLMGHCQKTMNQVVMPKQIVYWKQWLSLRNPILVFLKTRVLKRWRLFSKHE


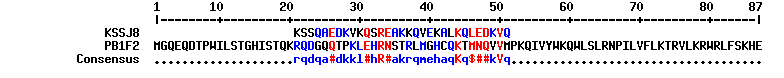


**KSS-J8 and Protein PA-X**

>KSSJ8

KSSQAEDKVKQSREAKKQVEKALKQLEDKVQ

>PAX

MEDFVRQCFNPMIVELAEKTMKEYGEDLKIETNKFAAICTHLEVCFMYSDFHFINEQGESIIVELGDPNALLKHRFEIIEGRDRTMAWTVVNSICNTTGAEKPKFLPDLYDYKENRFIEIGVTRREVHIYYLEKANKIKSEKTHIHIFSFTGEEMATKADYTLDEESRARIKTRLFTIRQEMASRGLWDSFVSPREEKRQLKKGLKSQEQCASLPTKVSRRTSPALKILEPMWMDSNRTATLRASCLKCPKK


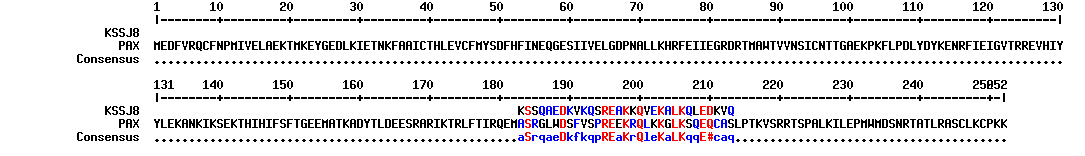

Supplement: Supplementary file 1 — Supplementary figures 1‐4 [file CTI2-10-e1337-s001.docx]
